# Assessment of newly designed fonts for visual accessibility

**DOI:** 10.1371/journal.pone.0345068

**Published:** 2026-03-24

**Authors:** Gordon E. Legge, Yingzi Xiong, Qingying Gao, Rachel Gage, Taylor Knickel, Charles Bigelow

**Affiliations:** 1 Department of Psychology, University of Minnesota, Minneapolis, Minnesota, United States of America; 2 Wilmer Eye Institute, Johns Hopkins University, Baltimore, Maryland, United States of America; 3 Department of Computer Science, Johns Hopkins University, Baltimore, Maryland, United States of America; 4 Cary Graphic Arts Collection, Rochester Institute of Technology, Rochester, New York, United States of America; University of Lahore - Raiwind Road Campus: The University of Lahore, PAKISTAN

## Abstract

**Purpose:**

The visual accessibility of fonts refers to the range of print sizes and efficiency with which readers can access text. One goal of font design may be to maximize accessibility for a wide range of users including those with low vision. Here, we compare behavioral and automated methods for evaluating the accessibility of a font for both normal and simulated low vision.

**Method:**

We evaluated the accessibility of a newly designed font, ACT Easy. In Experiment 1, we used a behavioral (psychophysical) approach to compare regular and bold versions of ACT Easy to Courier, Frutiger, and Gotham. 22 normally sighted young adults were tested with a computerized version of MNREAD in two conditions: normal viewing, and text digitally filtered to simulate moderate low vision (20/90 acuity). The outcome measures were reading acuity, critical print size, maximum reading speed, and participants’ preference rankings. In Experiment 2, we used an automated method to estimate the equivalent of reading acuity for eleven state-of-the-art Optical Character Recognition models. The models read MNREAD sentences in ACT Easy and five mainstream fonts. We explored how accurately the models mimicked human performance.

**Results:**

In Experiment 1, ACT Easy Regular compared well in reading acuity and critical print size with Courier, the best of the other fonts for both normal and simulated low-vision conditions. ACT Easy Regular and Gotham were favored in the preference rankings. In Experiment 2, nine of the eleven OCR models showed changes in reading acuity similar to humans in the normal and simulated low-vision conditions. Two of the models also exhibited human-like variations across fonts.

**Conclusions:**

Behavioral and automated methods are both capable of revealing subtle differences in the visual accessibility of fonts. The behavioral method requires labor-intensive human testing. The automated method does not require human testing, and may sometimes provide a practical alternative.

## Introduction

Thousands of fonts have been designed for rendering alphabetic text, but few of them have been evaluated empirically for their accessibility for people with normal or low vision. Low vision is any chronic form of vision impairment, not correctable by glasses or contacts, that affects everyday visual function. Estimates of the prevalence of vision impairment (including both blindness and low vision) are surprisingly high-- 7.08 million in the U.S. in 2017 [1], and 596.3 million worldwide in 2020 [2] with these numbers expected to rise rapidly in the future with the growing population of seniors. Almost everyone with low vision faces challenges with reading.

In this paper, the visual accessibility of text refers to the visual component of reading, that is, ability and efficiency in recognizing and reading printed characters in text. It encompasses the related concepts of legibility and readability [3] and also addresses the inclusiveness of text for individuals with a wide range of vision conditions. A broader definition of accessibility would include comprehension of the meaning of text as well, but comprehension is not included in the present analysis.

Many possible factors may play a role in optimizing text properties to enhance visual accessibility for both normal and low vision. Important factors include print size, luminance contrast, contrast polarity (black-on-white or white-on-black text), color, display size, layout of text on the page, and environmental conditions such as lighting and viewing distance. For a review, see Legge [4]. The intrinsic design properties of typefaces may affect accessibility as well, including details of shape and spacing of characters [5]. The visual accessibility of text may also depend on the type and strategy of reading, described in several ways: for low vision--spot, fluent and high fluent reading [6]; for normally sighted reading--scanning, skimming, rauding (i.e., typical reading of continuous text), learning and memorizing [7]; and more recently in reference to digital text--glanceable, interlude, and long-form reading [8]. Accessibility may also relate to the purpose of text, including its use in headlines or titles, body text, and signage.

It has been difficult to demonstrate major advantages of fonts designed specifically for low vision. Examples of specially designed fonts include Tiresias, developed at the Royal National Institute of the Blind in London, England [9], and APHont designed at the American Printing House for the Blind [10]. Despite various recommendations for font characteristics favorable for low vision, a review by Russell-Minda et al. [11] concluded that there is little empirical evidence for an optimally legible font for readers with low vision.

More recent examples of fonts designed specifically for low vision include Eido [12] and Maxular RX [13] intended for readers with central-vision loss from macular degeneration. In a study comparing reading performance of these two new fonts with Times, Courier and Helvetica for participants with normal vision and those with macular degeneration, the new fonts had slightly better reading acuity than Times or Helvetica, but no advantage over Courier [14].The authors of this study observed that there may be an advantage for some people with low vision in reading small print with fonts having larger inter-letter spacing relative to x-height. The font Luciole was designed for children and young adults with low vision. In a study, Galiano et al. [15] compared reading performance and eye movements with Luciole and five other fonts. They found subjective preference for Luciole, but no important differences in reading performance.

Fonts have also been designed for other special groups, especially those with reading disabilities. These include Open Dyslexia, EasyReading, and Dyslexi. Similar to the case of low vision, it has been difficult to find clear evidence for the advantages of these fonts over mainstream fonts [16–19]. But see Bachmann & Mengheri [20] who found an advantage for EasyReading over Times New Roman in a study of good readers and dyslexic children in fourth grade.

Given the difficulty in designing optimized fonts for groups with heterogeneous forms of low vision and reading disabilities, a practical goal, in keeping with the principle of inclusive design, may be to create fonts that achieve measures of good visual accessibility for readers with both normal and impaired vision.

This paper focuses on evaluating the accessibility of typefaces, with particular emphasis on the roles of print size and viewing distance. Most narrative text on screens or hard copy is read at a near viewing distance, with print size being a critical parameter. Most signage is viewed from afar where viewing distance becomes increasingly important.

**Fig 1:**
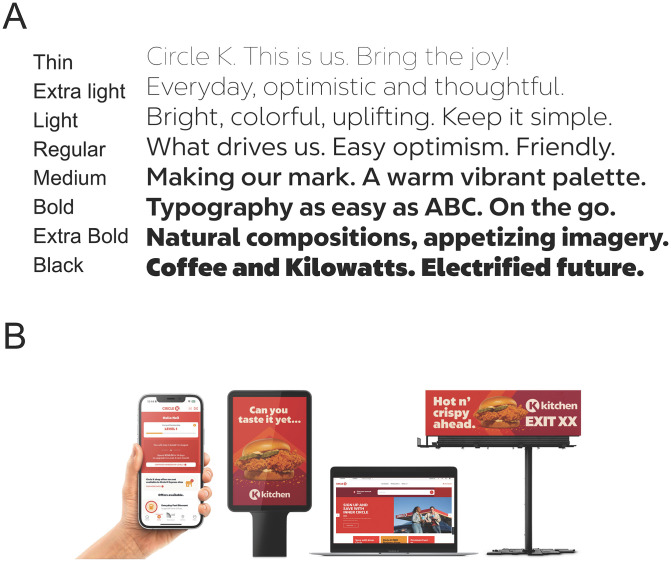
ACT easy font. Examples of ACT Easy (A) and its use in commercial applications (B). Use of this figure was approved by Isobel Cluskey, Director of Creative, Circle K Procurement and Global Brands.

We were asked by a typeface design company (Monotype) and their client (Circle K) to evaluate the visual accessibility of a new typeface intended for branding and commercial use. Circle K wanted a typeface with good visual accessibility for people with both normal and low vision, and wished to have evidence for the accessibility of their new typeface. The new typeface family is called ACT Easy, named after Alimentation Couche-Tard (ACT), the parent company of Circle K. It has regular, bold and italic versions, and a monospaced bold style for gas pump interfaces. Examples of ACT Easy are shown in Fig 1, along with its use in commercial applications.

Our study took place during the development of ACT Easy, focusing on comparing the reading accessibility of two versions of the newly designed typeface, ACT Easy Regular and ACT Easy Bold, with Courier, Gotham, and Frutiger. Gotham and Frutiger are representative of sans serif typefaces in geometric and humanist genres. Sans serif fonts are often recommended for accessibility, but may interact with other font features such as the ratio of thick to thin stroke widths within letter forms [21]. The design for the ACT Easy typeface also used elements from the geometric and humanist genres [22]. Courier was included because of its previous good performance in studies of low-vision accessibility [23,24].

In this paper, we describe two methods for evaluating the visual accessibility of a typeface, using ACT Easy as a case study. One method uses a traditional psychophysical laboratory approach with human participants, which we have used previously with both normal and low vision. We described this approach in a study of two typefaces designed specifically for people with macular degeneration [14]. In Experiment 1, we used a similar approach for evaluating ACT Easy.

The labor-intensive nature of the psychophysical lab approach may explain why there have been relatively few studies of typeface accessibility, especially for people with low vision. Some recent alternatives to lab-based human performance testing for normal vision may facilitate typeface evaluations, and include recruitment and testing of online participants (see Wallace et al. [8] for a crowd sourcing method), or cellphone-based mobile testing (see Dobres et al. [25]). Eye movement measures have also been employed to supplement psychophysical measures of reading performance in normal and low vision [15].

In Experiment 2, we explored an automated method for evaluation of the accessibility of typefaces for low vision which does not require direct human-performance testing. It involves filtering text to simulate reduced acuity and contrast sensitivity, followed by computer-based optical character recognition (OCR). Gao et al., [26] evaluated the performance of several OCR applications in recognizing letters, reading MNREAD sentences, and identifying text in complex environmental images across a range of print size. The stimuli were filtered to represent several levels of reduced acuity and contrast sensitivity, mimicking a range of low-vision conditions. The authors proposed that suitably chosen and fine-tuned OCR software may be helpful in predicting text accessibility for low vision. We examined this method for comparing ACT Easy with existing mainstream typefaces.

A brief note on terminology: The terms “typeface” and “font” are frequently used synonymously, but in this paper we use “typeface” when referring more to type designs, and “font” when referring more to functional aspects of type.

The two purposes of this report are 1) to illustrate two methods for evaluating the accessibility of a candidate font for both normal and low vision; and 2) to compare the performance of an automated approach with a psychophysical approach.

## Experiment 1: Psychophysical

The psychophysical method used for evaluating the visual accessibility of ACT Easy closely followed the method we used to evaluate fonts designed to facilitate reading in macular degeneration [14], and is described more briefly here.

### Methods – Experiment 1

#### Participants.

22 normally sighted adults participated in the main experiment (mean age 21.1 years, range 18−34; 3 male, 19 female). Data from one was excluded due to lack of English proficiency. The remaining 21 were native English speakers with no known nonvisual reading disabilities. Letter acuities ranged from −0.22 to 0.02 logMAR with a mean of −0.14 logMAR (Snellen equivalent 20/14.5). Participants were tested binocularly, wearing their presenting refractive corrections, if any.

The study was approved by the University of Minnesota Institutional Review Board and complied with the Declaration of Helsinki. Informed written consent was obtained from all participants.

#### Apparatus and stimuli.

Testing was conducted with sentences from a computerized version of the MNREAD Reading Acuity Test shown on a Apple Cinema LCD display (dimensions: 59.6 x 33.4 cm). Sentences were presented as high-contrast black letters on a white background (298.5 cd/m^2^).

Five fonts were used: Courier, Frutiger, Gotham, Act Easy Regular, and ACT Easy Bold. Letter size was defined as x-height (the height of lower case “x”) [27], and angular print size was designated by x-height in logMAR.

The sentences were selected from the algorithmic MNREAD sentence generator described by Mansfield et al. [28]. The sentences complied with the MNREAD language constraints (high-frequency vocabulary in U.S. third-grade text), and constraints on length (sixty characters per sentence), and layout (three single-spaced lines in a block of fixed aspect ratio). Sentences were chosen from the generator algorithm that simultaneously met these constraints for all of the five tested fonts.

**Fig 2:**
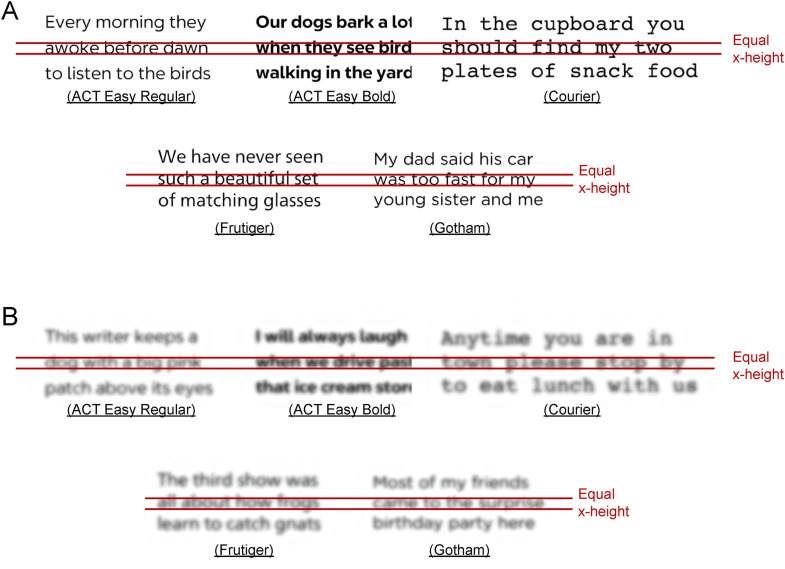
Fonts used in Experiment 1. Examples of unfiltered (A) and filtered (B) test sentences for the five fonts are shown. Two red lines mark the equal x-heights across all five fonts.

Testing was conducted with two conditions: normal viewing with unfiltered images, and filtered images to simulate acuity reduction to approximately 20/90, associated with mild low vision. Filtering was accomplished digitally, based on shifted versions of the human contrast sensitivity function to account for reduced acuity and contrast sensitivity [29], and shown to match the clinical acuities and contrast sensitivities of participants with low vision [30]. We will refer to the filtering condition as the simulated low-vision condition. Examples of filtered and unfiltered test sentences for the five fonts are shown in Fig 2.

#### Procedure.

For the normal (unfiltered) condition, reading speed was measured for each font at 13 print sizes, ranging from 0.9 to −0.3 logMAR in steps of 0.1 logMAR. Viewing distance was 40 cm for print sizes from 0.9 logMAR to 0.3 logMAR, and 160 cm for print sizes from 0.2 logMAR to −0.3 logMAR to ensure adequate pixel resolution of letters. For the simulated low-vision condition, the 13 print sizes ranged from 1.6 to 0.4 logMAR and were all tested at a viewing distance of 40 cm. We used different ranges of print sizes for the normal and simulated low-vision conditions to make sure that the reading speed would reach plateau.

The protocol and range of print sizes were refined through a first phase of preliminary testing on 15 participants using only mainstream fonts (results not detailed in Experiment 1 but included in Experiment 2 for model evaluation).

Testing followed the standard MNREAD protocol described by Mansfield & Legge [31] and summarized at http://MNREAD.umn.edu. In the test, participants were asked to read the sentences aloud as quickly and accurately as possible. The time to read each sentence was recorded, and the experimenter counted any errors. These measures were converted to reading speed in words per minute (wpm).

**Fig 3:**
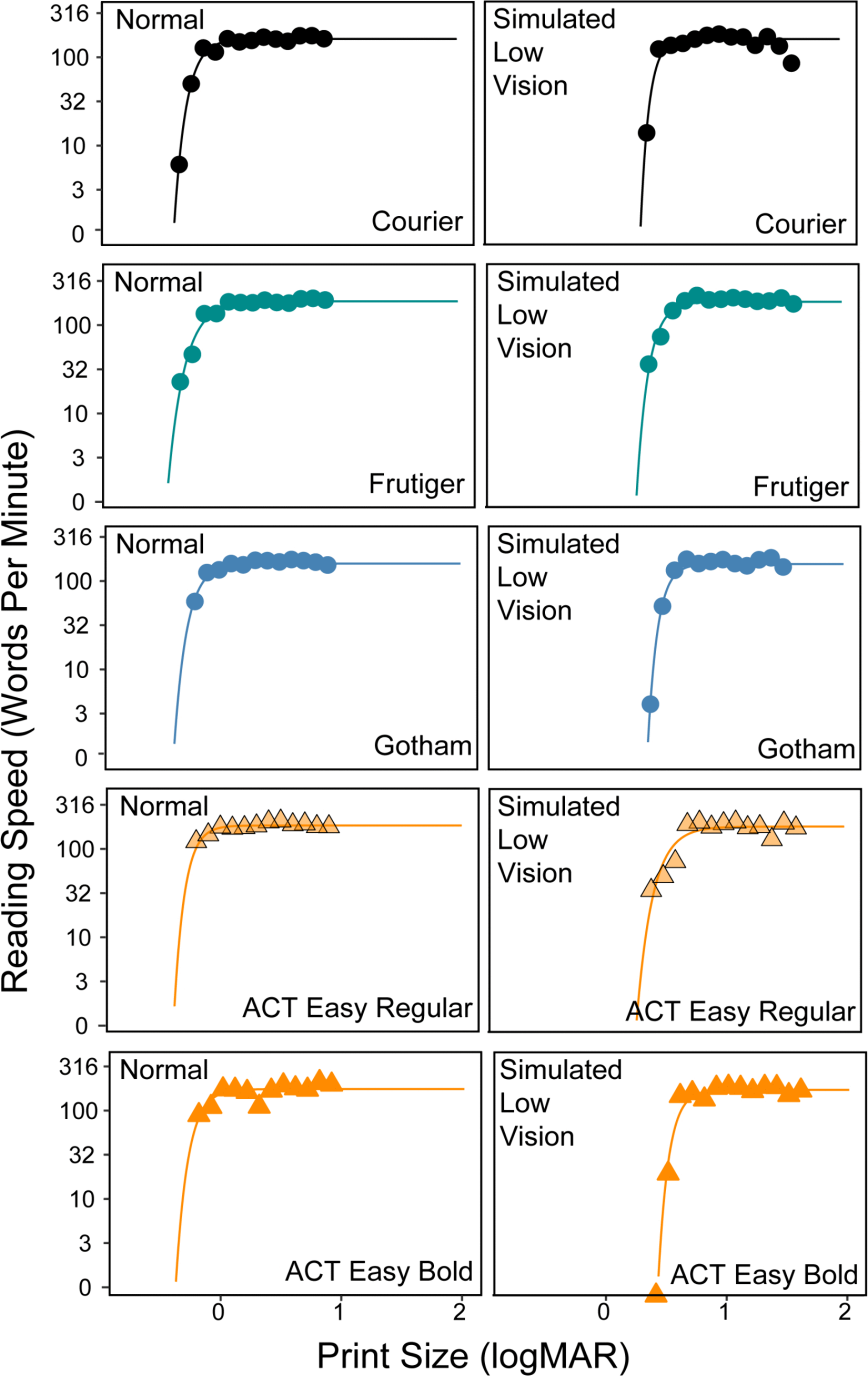
Results for one participant in Experiment 1. Reading curves showing reading speed (in words per minute) as a function of print size (in logMAR), for one sample participant. The left and right panels show reading curves under the normal and simulated low vision conditions. Each plot corresponds to one font, with the dots representing the empirically measured reading speed, and the curves showing fitted reading speed.

Reading speed was measured with two sentences at each of the 13 print sizes, five fonts, and both normal and simulated low-vision conditions, for a total of 260 trials. The normal condition was tested first, and font order and sentence sets were counterbalanced across participants. A participant’s performance for a given condition and font was a graph of reading speed as a function of print size. Examples are shown in Fig 3.

After all tests were completed, participants were asked to rank the fonts based on their preference, for the normal and simulated low-vision conditions separately. They were shown a hard copy sheet with the pangram “the quick brown fox …” printed in each of the five tested fonts. The highest ranking font was assigned a score of 5, and the lowest ranking font was assigned a score of 1.

In a session lasting between one and two hours, participants were tested with the five fonts in both normal and simulated low-vision conditions.

### Data analysis

Reading-speed versus print-size curves were fitted by an exponential function (fitted lines in Fig 3):


$$y=mrs\left(1-e^{-e^{lrc}}\left(x-xint\right)\right)$$

where y is log reading speed, mrs is the maximum reading speed (MRS), x is print size, lrc is the rate of change in reading speed, and xint is the print size at which reading speed is 0 log wpm.

Three parameters were obtained as measures of reading performance:

- Reading Acuity (RA): the smallest print size that can be read. Calculated as the smallest print size (in logMAR units) approached, penalized by number of word errors (number of word errors × 0.01).
- Critical Print Size (CPS): the smallest print size yielding best reading speed. Calculated as the print size corresponding to a reading speed of 95% of MRS. The CPS is often substantially larger than the RA, and is the target print size often prescribed for effective reading.
- Maximum Reading Speed (MRS): the fastest reading speed participants can achieve without any constraint on print size, and calculated as the plateau of the fitted exponential curve.

Our primary outcome measures were RA, CPS, MRS and rank-ordered font preference. Any or all of these four measures could be affected by variations in the choice of fonts, and also by vision status, in our case, normal and simulated low-vision conditions. Mean values and standard deviations were then obtained for the four reading parameters.

To quantitatively compare these parameters across fonts, we conducted linear mixed effect models on each of the parameters, with font and condition as fixed factors, and participant as random factors. Significant main effects of font and condition were examined using the “anova” function. Posthoc pair-wise comparisons were conducted with Bonferroni corrections using the “emmeans” package.

### Results – Experiment 1

**Fig 4:**
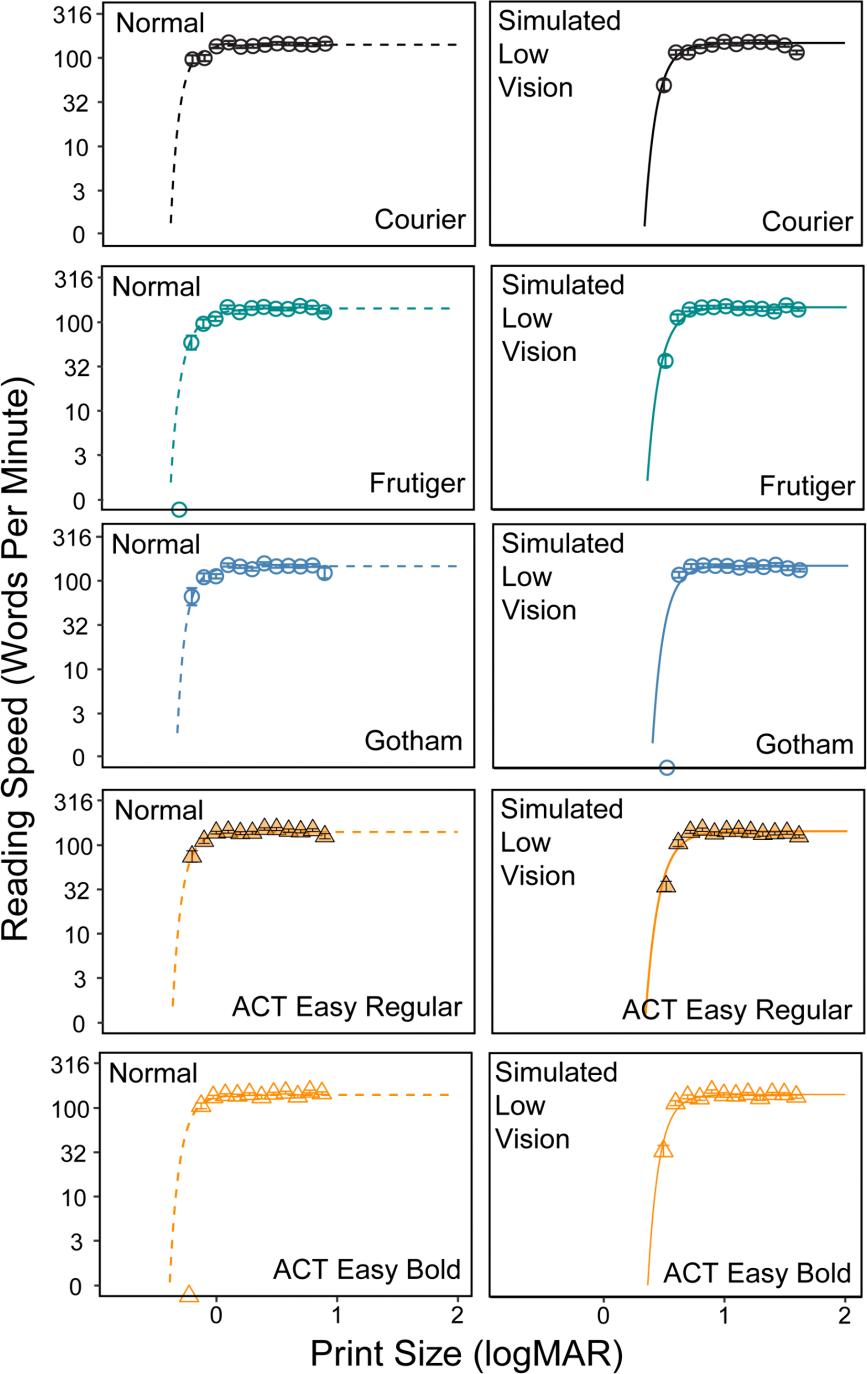
Reading results for Experiment 1. Reading curves showing reading speed (in words per minute) as a function of print size (in logMAR), means across all participants. The left and right panels show reading curves under the normal and simulated low vision conditions. Each plot corresponds to one font, with the dots representing the empirically measured reading speed, and the curves showing fitted reading speed. Error bars represent standard errors but are small and hard to distinguish from the data points.

**Fig 5:**
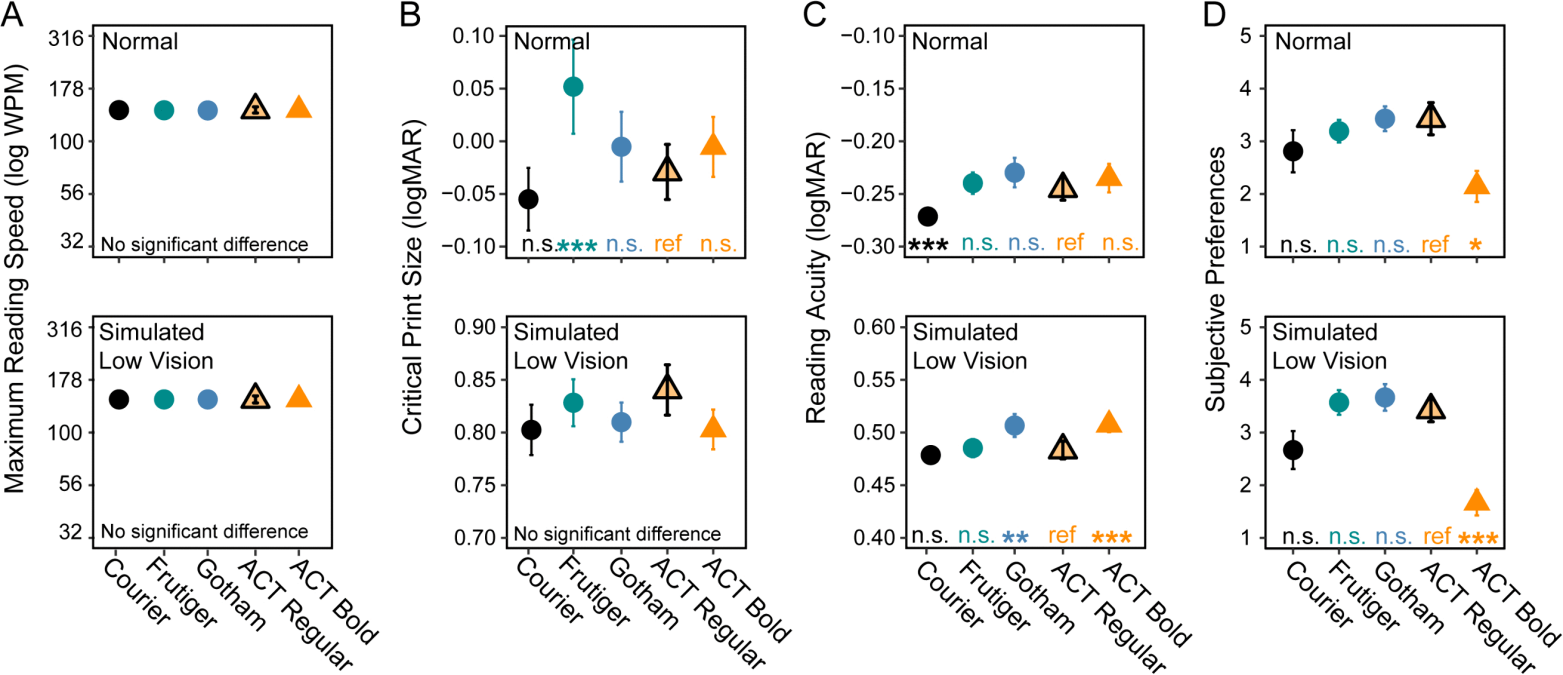
Summary of the four reading parameters across the five fonts. A. Maximum reading speed in words per minute. B. Critical print size in logMAR. C. Reading acuity in logMAR. D. Preference rating from 1-5. The upper and lower panel show results from the normal and simulated low vision conditions. In each plot, each symbol represents one of the five fonts. Error bars represent standard error across all participants.

Fig 4 shows the group averaged MNREAD curves. Reading speed in words per minute (wpm) is plotted on a logarithmic scale as a function of angular print size (logMAR). The five panels show results for the five fonts and two conditions. The exponential fits are shown by dashed lines (normal conditions), and solid lines (low-vision conditions). Table 1 lists means and standard deviations for the four measures—MRS, CPS, RA, and Preference Ranking. The two rows of Fig 5 plot corresponding means and standard deviations of the three reading measures and preference scores, normal condition in the upper row and simulated low-vision condition in the lower row. In the description below, we compare the performance of ACT Easy Regular to the other fonts because its properties were the primary focus of our study. In Fig 5, significant differences between ACT Easy Regular and other fonts are shown by asterisks above the x-axis.

**Table 1:** Means and standard deviations of reading measures.

Condition	Font	RA, logMAR Mean (SD)	CPS, logMAR Mean (SD)	MRS, wpm Mean (SD)	Preference, Mean (SD)
Normal	ACT Regular	−0.25 (0.05)	−0.03 (0.12)	142.43 (21.61)	3.43 (1.40)
	ACT Bold	−0.24 (0.06)	−0.01 (0.13)	142.37 (21.35)	2.14 (1.35)
	Courier	−0.27 (0.03)	−0.06 (0.14)	142.17 (21.53)	2.81 (1.83)
	Frutiger	−0.24 (0.05)	0.05 (0.21)	141.91 (21.35)	3.19 (0.98)
	Gotham	−0.23 (0.06)	−0.01 (0.15)	141.73 (21.72)	3.43 (1.08)
Simulated Low Vision	ACT Regular	0.48 (0.04)	0.84 (0.11)	145.71 (24.86)	3.43 (1.03)
	ACT Bold	0.51 (0.03)	0.80 (0.09)	145.71 (24.86)	1.67 (1.11)
	Courier	0.48 (0.03)	0.80 (0.11)	145.71 (24.86)	2.67 (1.65)
	Frutiger	0.49 (0.04)	0.83 (0.10)	145.71 (24.86)	3.57 (1.08)
	Gotham	0.51 (0.05)	0.81 (0.09)	145.71 (24.86)	3.67 (1.16)

The four measures of reading revealed the following:

Maximum Reading Speed: There was no significant difference in MRS across fonts (F(4, 180) = 0.80, p = 0.99). There was a significant but very small difference between the normal and simulated low-vision conditions by 0.01 log wpm (F(1, 180) = 37.22, p < 0.001). Maximum values were all quite close to 150 WPM.

Critical Print Size: The font effect was significant under the normal condition (F(4, 80) = 13.86, p < 0.001). Courier (the only fixed-width font) had the smallest (best) critical print size (−0.06 logMAR), ACT Easy the next smallest (−0.03 logMAR, p = 0.89), and Frutiger the largest (0.05 logMAR), but the extent of the entire range was small (0.11 logMAR). For the simulated low-vision condition, as would be expected, the CPS values are substantially larger (about 0.8 logMAR) than for the normal condition, but the range across the five fonts is very small (0.04 logMAR) without significant differences between fonts.

Reading Acuity: In the normal condition, Courier had the best reading acuity value (−0.27 logMAR), followed closely by ACT Easy Regular (−0.25 logMAR) and the other three fonts (all ps < 0.001). The range of RA values across fonts was very small (0.04 logMAR). It is noticeable that the RA value for Courier (−0.27 logMAR) is substantially better than the mean letter acuity for this group (−0.14 logMAR). It has been observed in past studies that reading acuity is better than letter acuity for normally sighted participants [32], likely due to benefits from lexical and sentence context in reading, and also differences in measurement procedures. For the simulated low-vision condition, ACT Easy Regular matched Courier with the lowest (best) values of RA (0.48 logMAR for both), and its advantage over Gotham and ACT Easy Bold were significant (p < 0.01). Again, the range of RA values was small.

Preference: Preference rankings ranged from 5 (best) to 1 (worst). The ratings were similar for the normal and simulated low-vision conditions. ACT Easy Regular, Gotham and Frutiger were favored over Courier and ACT Easy Bold (ps < 0.05).

To summarize the performance of ACT Easy Regular, it matched the comparison fonts in MRS, and approached Courier in CPS and RA. Compared to Gotham and ACT Easy Bold, ACT Easy Regular allowed a slightly smaller print size to be read under the simulated low-vision condition. ACT Easy Regular and Gotham were preferred by participants under both the normal and simulated low-vision conditions. When combining the reading accessibility and user preferences, ACT Easy Regular appeared to be the best performing font.

## Experiment 2: Computational

Gao et al. [26] proposed a method for automating assessment of the accessibility of text. They used OCR models to recognize acuity letters and MNREAD sentences across a range of print sizes. They evaluated the performance of both traditional OCR-specific models, and vision-language models (VLMs). The latter type, such as GPT 4o, were trained on scenes and also have text-recognition capabilities. Gao et al. compared their results with corresponding human data. They presented the OCR models with stimuli filtered to simulate reduced acuities in low vision. The primary measure of reading performance was reading acuity (RA), that is, the smallest print size that could be read. Reading speed was not part of the evaluation. The goal was to determine whether the OCR models would resemble human performance in response to changes in print size and acuity reduction. Any OCR model that closely mimics human performance would be a candidate for automated assessment of text accessibility. We used this approach in evaluating the accessibility of the fonts discussed in Experiment 1. Our purpose was to determine whether any of the OCR models would resemble the empirical findings from our human participants.

### Methods

The OCR models in our study were primarily large VLMs from major industry companies. SeeingAI, an app for assisting low vision and blind users was also included.

- GPT family (by OpenAI): GPT4o, and GPT4o_mini
- Gemini family (by Google): Gemini_15_pro, Gemini_15_flash, and Gemini_2_flash,
- Claude family (by Anthropic): Claude3_7_sonnet, and Claude3_5_haiku
- Qwen family (by Alibaba): Qwen2.5-VL-3B-Instruct, Qwen2.5-VL-7B-Instruct, and Qwen2.5-VL-32B-Instruct
- SeeingAI (by Microsoft)

### Apparatus and stimuli

The stimuli were the same images of MNREAD sentences used for testing human participants. Recognition performance was evaluated across a range of print sizes (calibrated in units of pixels per x-height) for unfiltered images (normal condition) and filtered images (low-vision condition corresponding to 20/90). Under the normal condition, the print sizes in x-height ranged from 2 to 19 pixels in steps of 0.1 log unit. Under the simulated low-vision condition, the print size ranged from 6 to 95 pixels in steps of 0.1 log unit.

The measure of reading accessibility for the OCR models was reading acuity. RA was computed using the same scoring rules used with human participants. For each OCR model, the print size in pixels per x-height was measured in the unfiltered (normal) condition and filtered (simulated low-vision) condition. For example, if an OCR model’s RA for unfiltered MNREAD sentences was 6 pixels per x-height and its reading acuity for the filtered sentences was 60 pixels per x-height, the difference would be a factor of ten in print size (one log unit). This change can be compared with the difference in average logMAR reading acuities for human participants between normal and corresponding low-vision conditions.

We conducted this analysis for the seven fonts -- Courier, Frutiger, Gotham, Helvetica, Times, ACT Easy Regular, and ACT Easy Bold. For each viewing condition (normal and simulated low vision), font, and OCR model, we computed mean RA from two MNREAD sentence sets. We evaluated each OCR model once for every text sample, but the results are repeatable because the models produce the same outputs when given the same input parameters and when setting the noise parameter (temperature) to zero. Qwen series is open source, so with a fixed model size, the architecture and trained weights do not change. Although GPT, Claude, and Gemini are closed source, we used the versions released on specific dates for reproducibility.

### Results – Experiment 2

Our primary question was to determine whether image filtering to simulate reduced acuity in low vision would lead to the same change in reading acuity (measured in log units) as observed with human participants. We were also interested to see if the OCR models behaved differently across fonts, and whether such variations would mimic human performance.

**Fig 6:**
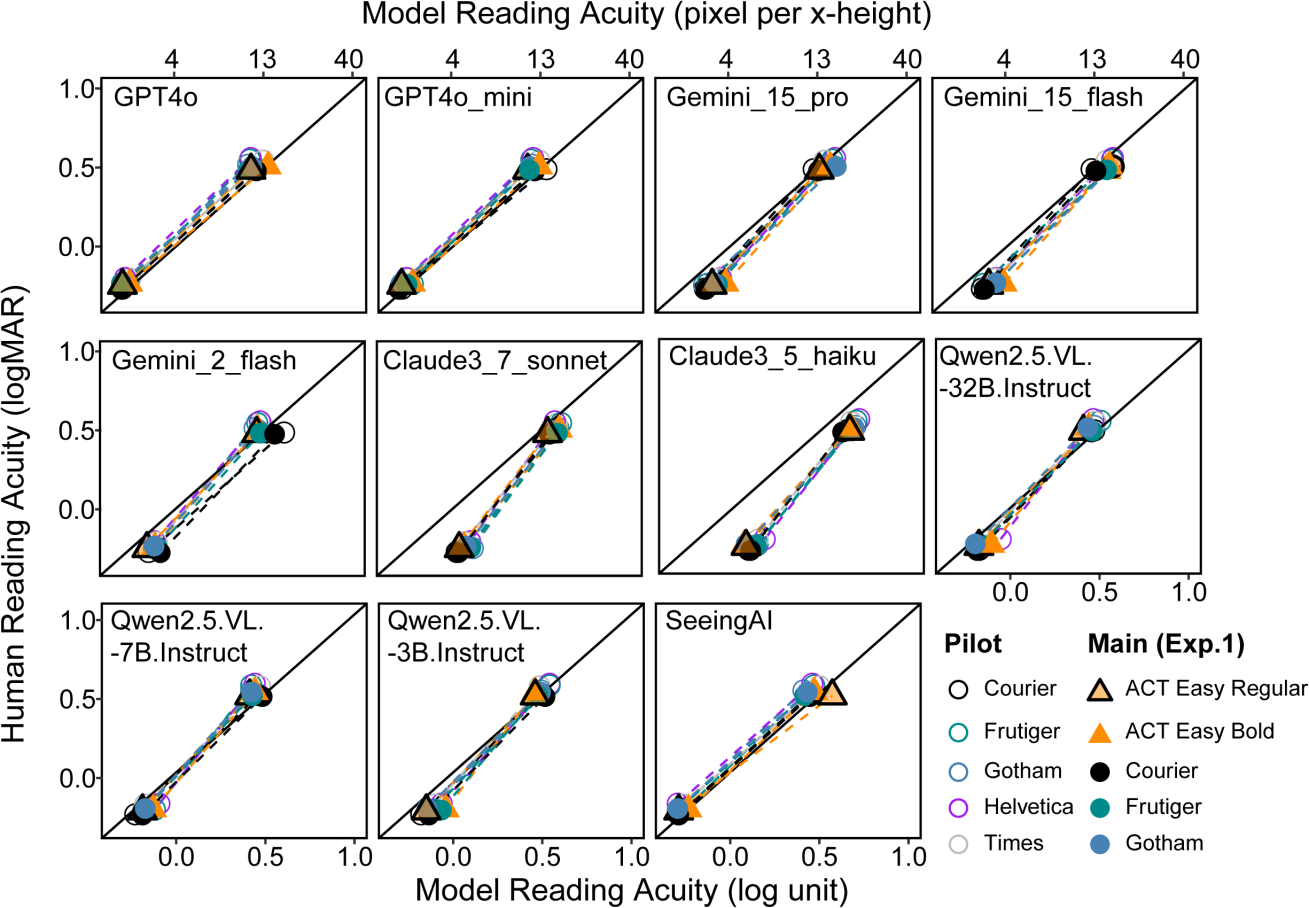
Comparison of model and human reading acuity. The bottom x-axis marks model reading acuity in log units, and the top x-axis marks the corresponding pixels per x-height, with 0 log unit corresponding to 4 pixels per x-height. Each panel represents a model. Each symbol represents a font. Pairs of symbols joined by dashed lines show performance in the normal and simulated low-vision conditions.

Fig 6 compares changes in reading acuity for the OCR models with changes in human reading acuity for the normal and simulated low-vision conditions. Each panel represents an OCR model, and each type of symbol represents a font. Dashed lines lying on the diagonal represent a good match between the OCR model and human performance. Note that the human RA came from two phases of testing-- pilot testing with only mainstream fonts, and the main experiment also including ACT Easy (results in Experiment 1). Because different participants participated in these two phases, we used solid and hollow symbols for these two phases in the plot. Most of the models performed well, showing very similar acuity changes to humans. The exceptions are the two Claude models. The Claude models had much worse acuity in the normal condition, needing more pixels per x-height for correct word recognition. Although their performance in the simulated low-vision condition was comparable to other OCR models, the differences between normal and simulated low vision were smaller.

**Fig 7:**
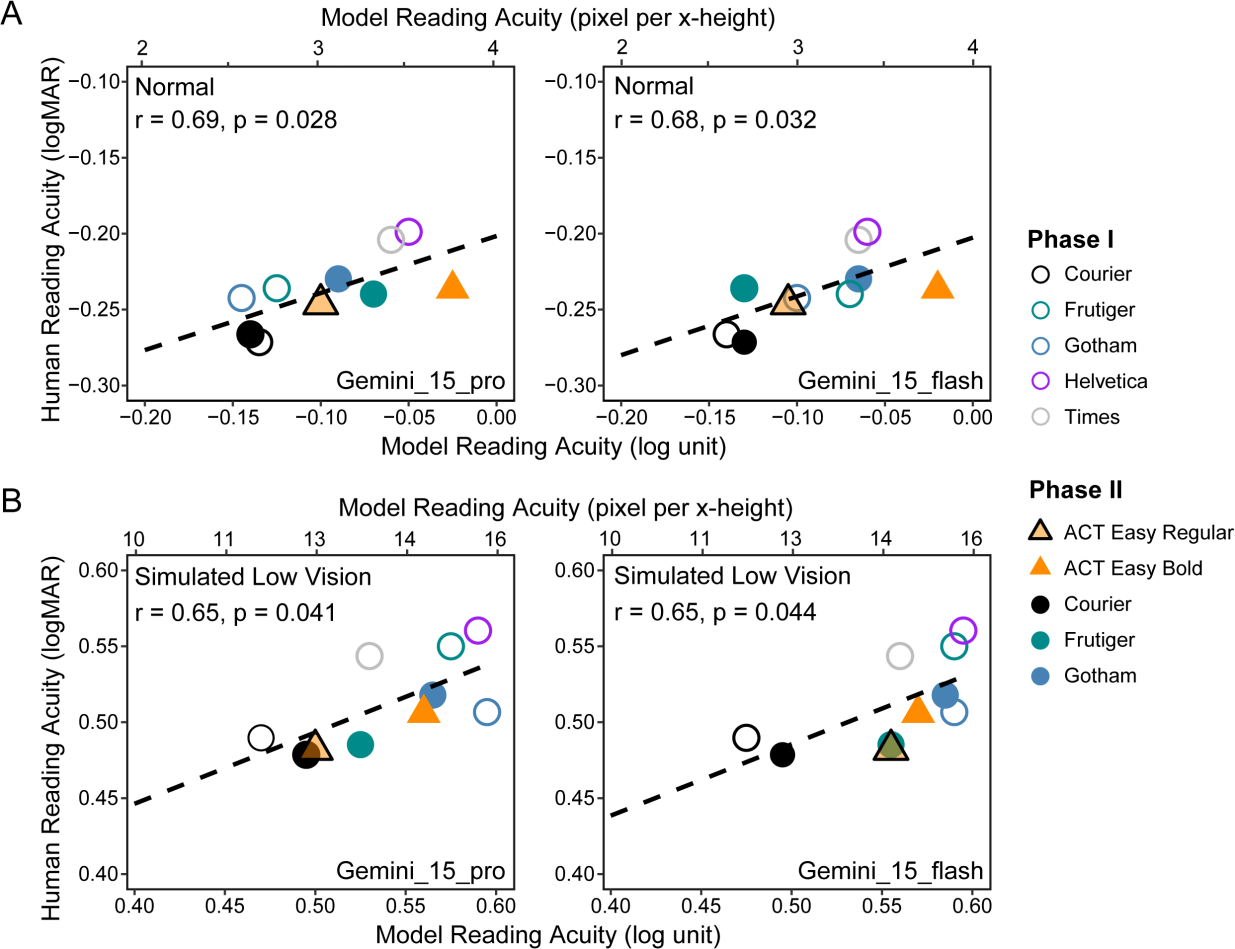
Comparison of acuity for humans and two Gemini models. Comparison of model and human reading acuity under the normal (A) and simulated low vision (B) conditions, for Gemini_15_pro (left) and Gemini_15_flash (right). The bottom x-axis marks model reading acuity in log units, and the top x-axis marks the corresponding pixels per x-height, with 0 log unit corresponding to 4 pixels per x-height. Each symbol represents a font. The hollow symbols represent data from the pilot study, and the solid symbols represent data from the main study. Dashed lines are the regression lines across all fonts. The correlation results are also provided with each plot.

Next, we asked whether the OCR models would resemble human variations in RA across fonts. Table 2 summarizes the correlation results between each OCR model and the human RAs. Only the two Gemini models (Gemini_15_pro and Gemini_15_flash) showed significant correlations with human participants in both normal and simulated low-vision conditions (Fig 7). In both conditions, the magnitude of variations in RA across fonts was similar for the OCR models and human participants, with the difference between best and worst fonts being close to 0.1 log unit.

**Table 2:** Correlation between OCR and human reading acuities across fonts.

	Normal		Simulated low vision	
Model	Correlation coefficient	P value	Correlation coefficient	P value
GPT4o	0.53	0.116	−0.05	0.899
GPT 4o_mini	0.51	0.133	0.00	0.996
Gemini_15_pro	**0.69**	**0.028**	**0.68**	**0.032**
Gemini_15_flash	**0.65**	**0.041**	**0.65**	**0.044**
Gemini_2_flash	0.18	0.618	−0.35	0.323
Claude3_7_sonnet	0.58	0.079	**0.64**	**0.048**
Claude3_5_haiku	0.58	0.082	**0.76**	**0.011**
Qwen2.5-VL-32B-Instruct	0.62	0.054	0.59	0.073
Qwen2.5-VL-7B-Instruct	**0.67**	**0.036**	−0.05	0.885
Qwen2.5-VL-3B-Instruct	0.57	0.087	0.61	0.061
SeeingAI	0.21	0.552	0.07	0.853

Values in bold are statistically significant.

What do these results tell us about the ability of the OCR models to mimic human RA across viewing conditions (normal and simulated low vision) and fonts? The Gemini series does best among the models, and reliably replicated the difference in viewing conditions and fonts. Both Gemini and humans showed better RA of Courier than Helvetica, Times and most other fonts, a finding consistently reported in previous font studies. Further, both Gemini and humans showed that ACT Easy Regular, a typeface Monotype designed for Circle K, approaches Courier in acuity in both normal and low-vision conditions and outperforms other mainstream fonts.

These results support the plausibility of using an automated OCR method to evaluate the impact of reduced acuity in low vision on reading accessibility and also the fine-grained effects of typeface design.

## Discussion

The original motivation for this study was to evaluate the visual accessibility of the newly designed typeface ACT Easy. We measured reading performance of normally sighted participants who read text sentences across a wide range of print sizes with normal viewing and with text filtered to simulate acuity reduction associated with mild low vision. We compared reading measures and preference for ACT Easy with the mainstream fonts Courier, Gotham and Frutiger. There were four measures—Reading Acuity, Critical Print Size, Maximum Reading Speed, and preference ranking.

### Performance of the new font ACT easy

Overall, the variations of the reading measures across fonts were small. This is not surprising, given the prior research literature. Mainstream fonts are widely used because they are highly legible for normally sighted readers. Gotham and ACT Easy Regular both performed well within this small range. They compared well with Courier, which has typically exhibited particularly good CPS and RA for people with low vision [23,24]. The advantage of Courier appears to be related to wide inter-letter spacing [14].

We can think of RA and CPS as the smallest print sizes that can be read. But we can also think of these measures as indicators of the distance at which print of any given size can be read. A font with good RA or CPS is likely to be legible at a greater viewing distance than a font with poorer RA or CPS. In commercial applications, the distance at which text can be read may be important. Even a small difference of 0.1 logMAR in RA or CPS translates into a difference in legible viewing distance of about 20 percent.

We also included preference ratings. Font preference can be affected by a variety of factors including print size as reported by Wallace et al. [8], but these authors did not find an effect of familiarity. We included preference because it might play a role in commercial applications. Our sample of normally sighted participants gave high rankings to ACT Easy Regular in both the normal and simulated low-vision formats.

To briefly summarize, the new font ACT Easy performed well in our measures of visual reading performance. Measured values compare well with values from existing mainstream fonts. In particular, ACT Easy Regular exhibited good reading acuity and critical print size, meaning that it would be accessible from viewing distances comparable and sometimes greater than other mainstream proportionally-spaced fonts. ACT Easy Bold had a slight disadvantage when compared to ACT Easy Regular in the objective reading parameters, and was least favored by the readers.

### Two methods for evaluating the visual accessibility of fonts

Given the proliferation of new fonts and the goal of designers to create fonts for special groups, such as people who are visually impaired, aging, or dyslexic, it would be helpful to have multiple methods for assessment of reading accessibility. We have described two methods in this paper, both potentially able to identify small differences in key measures of reading vision. The psychophysical method in Experiment 1 has been used previously in evaluating specially designed fonts for low vision [14], and similar methods have been used in other studies of low-vision reading. This approach can be effective, but is labor-intensive. It requires careful calibration of equipment and stimuli, and recruitment and testing of human participants who are tested in controlled settings.

The second method relies on the output of optical character recognition (OCR) models to mimic human performance, at least for assessment of reading acuity. If proven to provide valid results across a range of testing scenarios, this approach could automate the assessment of reading accessibility. The results described in Experiment 2 showed that some existing OCR models were able to mimic the human results we found in Experiment 1. In particular, most of the models we evaluated showed similar overall changes in RA between normal and simulated low vision conditions, but only two of the models showed similar effects of font variations. Evaluation of subtle font differences appeared to require more specialized OCR models.

If an OCR model were to become a valid guide for predicting reading accessibility, we are left with the challenge of understanding why the model behaves like humans. Perhaps this is similar to the ongoing challenge of understanding how large language models compare with humans in language understanding and information gathering. Most of the advanced OCR models have algorithms that are proprietary. In most cases, they have been trained on text that is legible for normally sighted human readers, and might be expected to learn to behave similarly to human readers. In the paper by Gao et al. [26] which motivated Experiment 2, the authors raise the possibility of enhancing OCR models for reading accessibility by added training with text filtered to simulate low-vision conditions.

## References

[1] Flaxman AD, Wittenborn JS, Robalik T, Gulia R, Gerzoff RB, Lundeen EA, et al. Prevalence of visual acuity loss or blindness in the US: a bayesian meta-analysis. JAMA Ophthalmol. 2021;139(7):717–23. doi: 10.1001/jamaophthalmol.2021.0527 33983373 PMC8120442

[2] GBD 2019 Blindness and Vision Impairment Collaborators, Vision Loss Expert Group of the Global Burden of Disease Study. Trends in prevalence of blindness and distance and near vision impairment over 30 years: an analysis for the Global Burden of Disease Study. Lancet Glob Health. 2021;9(2):e130–43. doi: 10.1016/S2214-109X(20)30425-3 33275950 PMC7820390

[3] Tinker MA. Legibility of print. Ames: Iowa State University Press; 1963.

[4] Legge GE. Psychophysics of reading in normal and low vision. Boca Raton: Routledge, Taylor & Francis Group; 2007.

[5] Bigelow C. Typeface features and legibility research. Vision Res. 2019;165:162–72. doi: 10.1016/j.visres.2019.05.003 31078662

[6] Whittaker SG, Lovie-Kitchin J. Visual requirements for reading. Optom Vis Sci. 1993;70(1):54–65. doi: 10.1097/00006324-199301000-00010 8430009

[7] Carver RP. Reading rate: a review of research and theory. San Diego: Academic Press. 1990.

[8] Wallace S, Bylinskii Z, Dobres J, Kerr B, Berlow S, Treitman R, et al. Towards individuated reading experiences: different fonts increase reading speed for different individuals. ACM Trans Comput-Hum Interact. 2022;29(4):1–56. doi: 10.1145/3502222

[9] Font T. MyFonts by Monotype Internet. 2001. Accessed 2026 January 28. https://www.myfonts.com/collections/tiresias-font-bitstream/?tab=techSpecs

[10] Kitchel JE. Large print: Guidelines for optimal readability and APHontTM a font for low vision. Louisville, KY: The American Printing House for the Blind, Inc.; 2004. http://www.aph.org/edresearch/lpguide.htm

[11] Russell-Minda E, Jutai JW, Strong JG, Campbell KA, Gold D, Pretty L. The legibility of typefaces for readers with low vision: a research review. J Vis Impair Blind. 2007;101(7):402–15. doi: 10.1177/0145482X0710100703

[12] Bernard J-B, Aguilar C, Castet E. A new font, specifically designed for peripheral vision, improves peripheral letter and word recognition, but not eye-mediated reading performance. PLoS One. 2016;11(4):e0152506. doi: 10.1371/journal.pone.0152506 27074013 PMC4830533

[13] Letters between friends: How a shared admiration for font design is helping people read again. UofL Magazine. 2024. Accessed 2026 January 28. https://news.louisville.edu/news/letters-between-friends-how-shared-admiration-font-design-helping-people-read-again

[14] Xiong Y-Z, Lorsung EA, Mansfield JS, Bigelow C, Legge GE. Fonts designed for macular degeneration: impact on reading. Invest Ophthalmol Vis Sci. 2018;59(10):4182–9. doi: 10.1167/iovs.18-24334 30128489 PMC6100668

[15] Galiano AR, Augereau-Depoix V, Baltenneck N, Latour L, Drissi H. Luciole, a new font for people with low vision. Acta Psychol (Amst). 2023;236:103926. doi: 10.1016/j.actpsy.2023.103926 37137180

[16] Rello L, Baeza-Yates R. The effect of font type on screen readability by people with dyslexia. ACM Trans Access Comput. 2016;8(4):1–33. doi: 10.1145/2897736

[17] Wery JJ, Diliberto JA. The effect of a specialized dyslexia font, OpenDyslexic, on reading rate and accuracy. Ann Dyslexia. 2017;67(2):114–27. doi: 10.1007/s11881-016-0127-1 26993270 PMC5629233

[18] Kuster SM, van Weerdenburg M, Gompel M, Bosman AM. Dyslexie font does not benefit reading in children with or without dyslexia. Ann Dyslexia. 2018;68(1):25–42. doi: 10.1007/s11881-017-0154-6 29204931 PMC5934461

[19] Powell SL, Trice AD. The impact of a specialized font on the reading performance of elementary children with reading disability. Contemp Sch Psychol. 2020;24(1):34–40. doi: 10.1007/s40688-019-00225-4

[20] Bachmann C, Mengheri L. Dyslexia and fonts: is a specific font useful?. Brain Sci. 2018;8(5):89. doi: 10.3390/brainsci8050089 29757944 PMC5977080

[21] Minakata K, Eckmann-Hansen C, Larsen M, Bek T, Beier S. The effect of serifs and stroke contrast on low vision reading. Acta Psychol (Amst). 2023;232:103810. doi: 10.1016/j.actpsy.2022.103810 36563495

[22] Shaw P. Revival type. New Haven: Yale University Press; 2017. 209–34.

[23] Mansfield JS, Legge GE, Bane MC. Psychophysics of reading. XV. Font effects in normal and low vision. Invest Ophthalmol Vis Sci. 1996;37(8):1492–501. 8675391

[24] Tarita-Nistor L, Lam D, Brent MH, Steinbach MJ, González EG. Courier: a better font for reading with age-related macular degeneration. Can J Ophthalmol. 2013;48(1):56–62. doi: 10.1016/j.jcjo.2012.09.017 23419299

[25] Dobres J, Klarl K, Kindelsberger J, Reimer B. TypeTester: a case study of behavioral data collection using a smartphone platform. MIT AgeLab; 2018. https://agelab.mit.edu/static/uploads/typetester-case-study.pdf

[26] Gao Q, Manduchi R, Ramulu PY, Legge GE, Xiong Y. VI-OCR: “Visually Impaired” optical character recognition pipeline for text accessibility assessment. Sci Rep. 2025;16(1):1269. doi: 10.1038/s41598-025-30982-7 41372330 PMC12789057

[27] Legge GE, Bigelow CA. Does print size matter for reading? A review of findings from vision science and typography. J Vis. 2011;11(5). doi: 10.1167/11.5.8 21828237 PMC3428264

[28] Mansfield JS, Atilgan N, Lewis AM, Legge GE. Extending the MNREAD sentence corpus: computer-generated sentences for measuring visual performance in reading. Vision Res. 2019;158:11–8. doi: 10.1016/j.visres.2019.01.010 30731097 PMC6538455

[29] Chung STL, Legge GE. Comparing the shape of contrast sensitivity functions for normal and low vision. Invest Ophthalmol Vis Sci. 2016;57(1):198–207. doi: 10.1167/iovs.15-18084 26795826 PMC4727522

[30] Xiong Y-Z, Lei Q, Calabrèse A, Legge GE. Simulating Visibility and Reading Performance in Low Vision. Front Neurosci. 2021;15:671121. doi: 10.3389/fnins.2021.671121 34290578 PMC8287255

[31] Mansfield JS, Legge GE. The MNREAD acuity chart. In: Legge GE, ed. Psychophysics of reading in normal and low vision. Boca Raton: Routledge, Taylor & Francis Group; 2007. 167–91.

[32] Xiong Y-Z, Calabrèse A, Cheong AMY, Legge GE. Reading acuity as a predictor of low-vision reading performance. Invest Ophthalmol Vis Sci. 2018;59(12):4798–803. doi: 10.1167/iovs.18-24716 30347073 PMC6181187

